# French national diagnosis and care protocol (Protocole National De Diagnostic et de Soins; PNDS): Gaucher disease

**DOI:** 10.1186/s13023-025-03980-1

**Published:** 2025-10-27

**Authors:** Fabrice Camou, Christine Serratrice, Magali Pettazzoni, Yann Nadjar, Anaïs Brassier, Soumeya Bekri, Bérengère Cador-Rousseau, Louis Dagneaux, Florence Dalbies, Roseline Froissart, Delphine Genevaz, Anne-Sophie Guemann, Bénédicte Hivert, Vanessa Leguy-Seguin, Catherine Marcel, Agathe Masseau, Martin Michaud, Yves-Marie Pers, Jérôme Stirnemann, Sabrina Vergnaud, Yann Nguyen, Catherine Caillaud, Samia Pichard, Marc G. Berger, Nadia Belmatoug

**Affiliations:** 1 Service de Médecine Interne, CHU de Bordeaux, Hôpitaux Haut Lévêque, Pessac, France; 2 Département de Médecine Interne de L’âgé, Hôpitaux Universitaires de Genève, Thonex, Suisse; 3 Laboratory of Biochemistry and Molecular Biology UM Inherited Metabolic and Red Blood Cell Diseases Hospices Civils de Lyon, Hospital Cluster East, Biology Center East, 69677 Bron Cedex, France; 4 Hospital Pitié Salpêtrière Department of Neurology Neuro-Metabolism, Assistance Publique-Hôpitaux de Paris, Filière G2m, 75013 Paris, France; 5 Centre de Référence Des Maladies Héréditaires du Métabolismes, Hôpital Necker-Enfants Malades, Assistance Publique-Hôpitaux de Paris, IHU Institut Imagine, Filière G2m, Paris, France; 6 Laboratory of Metabolic Biochemistry, Rouen University Hospital, 76000 Rouen, France; 7 Department of Internal Medicine, Pontchaillou University Hospital, 35033 Rennes Cedex 9, France; 8 Service d’Orthopédie, CHU de Montpellier, Montpellier, France; 9 Institut de Cancéro-Hématologie, CHRU Morvan, Brest, France; 10 Association VML, Vaincre Les Maladies Lysosomales, Paris, France; 11 Centre de Référence Des Maladies Héréditaires du Métabolisme de L’enfant Et de L’adulte, CHRU de Lille, Lille, France; 12 Service d’Hématologie, Hôpital Saint Vincent de Paul, Lille, France; 13 Service de Médecine Interne Et Immunologie Clinique, CHU Bocage Central, Dijon, France; 14 Service de Médecine Interne, CHU Hôtel Dieu, Nantes, France; 15 Department of Internal Medicine, Joseph Ducuing Hospital, BP 53160, 31027 Toulouse Cedex 3, France; 16 IRMB, Université de Montpellier, Inserm U1183, CHU Montpellier, Montpellier, France; 17 Service de Médecine Interne, Hôpitaux Universitaires de Genève, Geneva, Switzerland; 18 Rare Enzyme Inherited Diseases – CGD, SB2TE - IBP Grenoble Alpes University Hospital, CS 10217, 38043 Grenoble Cedex 9, France; 19 Service de Médecine Interne, Centre de Référence Des Maladies Lysosomales, Hôpital Beaujon, Université Paris Cité, Filière G2m, AP-HP NordClichy, France; 20 Centre for Research in Epidemiology and Statistics (CRESS), Université Paris Cité and Université Sorbonne Paris Nord, Inserm, INRAE, Paris, France; 21 Laboratory of Metabolic Biochemistry, Necker Hospital - Sick Children, 75015 Paris, France; 22 Centre de Référence Des Maladies Héréditaires du Métabolismes, Hôpital Necker-Enfants Malades, Assistance Publique-Hôpitaux de Paris, IHU Institut Imagine, Filière G2m, Paris, France; 23 Department of Biological Hematology, Center for Biological Resources (CBR) Auvergne, Estaing University Hospital, 63003 Clermont-Ferrand Cedex 1, France; 24 Service de Médecine Interne, Centre de Référence Des Maladies Lysosomales, Hôpital Beaujon, Université Paris Cité, 100 Bd du Général Leclerc, AP-HP92110 NordClichy, France

**Keywords:** Gaucher disease, Glucocerebrosidase deficiency, Enzyme replacement therapy, Substrate reduction therapy, Diagnosis, Monitoring, Lysosomal disease

## Abstract

Gaucher disease (GD) is a rare autosomal recessive lysosomal disorder caused by glucocerebrosidase deficiency, with a prevalence in France of around 1/130,000 people. The clinical picture of GD is very heterogeneous, ranging from lifelong asymptomatic forms to severe forms with onset during childhood, such as GD type 2 (< 1% of cases). GD type 1, the most common form (95% of cases), manifests with varying degrees of organomegaly, cytopenia and bone manifestations. Progressive encephalopathy of varying severity is also observed in GD type 3. Symptoms may result in acute and/or chronic pain and asthenia, and lead to disability. The aim of the French National Diagnosis and Care Protocol (Protocole National de Diagnostic et de Soins; PNDS) is to provide health care professionals with guidance for the optimal management and care of patients with GD. GD diagnosis is usually based on laboratory analyses revealing low or absent glucocerebrosidase activity, and can be confirmed by identification of glucocerebrosidase (*GBA1*) gene pathogenic variants. Additional assessments should include biological analyses (hemogram test, serum protein electrophoresis, and measurement of GD biomarkers), imaging examinations (X-rays, abdominal and bone magnetic resonance imaging, bone densitometry, echocardiography), and electrocardiogram.

Patient management in France is multidisciplinary and should be coordinated by a GD specialist, in conjunction with the Committee for the Evaluation and Treatment of Gaucher Disease, the Reference Center for Lysosomal Diseases or a reference/competence center for inherited metabolic diseases. The indication for treatment is not systematic and is based on the presence of clinical, biological, and imaging criteria. Current treatments such as intravenous enzyme replacement therapy or oral substrate reduction therapy, generally lead to significant improvements in disease characteristics within one to five years and early initiation can prevent complications. Follow-up should include a clinical examination, biological analyses to monitor disease biomarkers twice a year then yearly for stable patients, and imaging evaluations initially every year and then every 3 to 4 years for patients with stable disease in whom therapeutic objectives have been achieved. Intercurrent pathologies can be managed by the attending physician in collaboration with a GD specialist.

## Summary for the attending physician

### Background

Gaucher disease (GD) is an autosomal recessive lysosomal disorder caused by glucocerebrosidase deficiency. The prevalence of GD in France is around 1/130,000 people. In 2022, 521 individuals living with GD (446 adults and 75 children) were registered in the French Gaucher Disease Registry (Registre Français de la Maladie de Gaucher; RFMG). Clinical expression of GD varies, and diagnosis can be made at any age. The prescription of specific treatments for GD, such as enzyme replacement therapy (ERT) or substrate reduction therapy (SRT), is not systematic. Indications for therapy must be validated by a multidisciplinary group of experts, based on clinical, biological, and imaging criteria. Regular monitoring (clinical examinations, biological analyses, and imaging evaluations) is essential.

### Initial assessment

The initial assessment for GD should be multidisciplinary and coordinated by a GD specialist, in conjunction with the treating physician.

#### Clinical presentation

There are three main clinical forms of GD:

- Gaucher disease type 1 (GD1; OMIM#230,800, ORPHA77259) accounts for 95% of cases. In France, the median age of onset is 15 years and the median age at diagnosis is 22 years. Its clinical expression is very heterogeneous, ranging from forms that remain asymptomatic throughout life to severe forms with onset during childhood. GD1 may be associated with varying degrees of organomegaly (splenomegaly and hepatomegaly), cytopenia (thrombocytopenia, anemia and, more rarely, leukopenia) and bone damage (infarcts, avascular osteonecrosis, fractures, and osteopenia), which may result in acute and/or chronic pain and asthenia, and lead to disability. To avoid confusion, and because of different terminology is used by international teams with expertise in GD to name bone events, the French group defined a bone infarct as a an ischemic (or vaso-occlusive) event occurring in a long or a flat bone and osteonecrosis as an event occurring in an epiphysis.
- Gaucher disease type 2 (GD2; OMIM#230,900 and #608,013, ORPHA77260) is a very rare form (accounting for less than 1% of cases). It is associated with very early onset (before the age of 1 year) and a poor prognosis (death before the age of 3 years).
- Gaucher disease type 3 (GD3; OMIM#23,100 and #231,005, ORPHA77261) accounts for less than 5% of cases. In addition to systemic symptoms similar to those seen in GD1, GD3 is associated with progressive encephalopathy of varying severity (ocular motor anomalies, epilepsy, and ataxia). Onset occurs before the age of 20 years.

#### Positive diagnosis

Diagnosis of GD is based on the results of a blood test carried out at a reference biomedical laboratory (see Appendix 1) and demonstrating reduced glucocerebrosidase activity. Genetic testing can also be performed to confirm the diagnosis, identify the glucocerebrosidase (GBA1) gene pathogenic variants and characterize the genotype. Patients should also be asked whether they consent to being included in the RFMG (See Appendix 2) and in the national Longitudinal Study of Intrinsic and Extrinsic Determinants of Gaucher Disease (Etude LOngitudinale des Déterminants Intrinsèques et Extrinsèques de la Maladie de Gaucher; ELODIE-MG) (see Appendix 3).

#### Additional examinations

After diagnosis, additional assessments (see Appendix 4) should include biological analyses such a hemogram test, serum protein electrophoresis, and measurement of GD biomarkers (LysoGL1, chitotriosidase, and CCL18); and imaging examinations including a chest X-ray, abdominal magnetic resonance imaging (MRI) or ultrasound, electrocardiogram, echocardiography, skeletal X-rays, bone MRI (rachis, pelvis, femurs, and tibiae), and bone densitometry.

### Therapeutic management

As GD is designated as a chronic health condition in France, the cost of managing patients diagnosed with GD (diagnostic tests, treatment, monitoring, etc.) are covered by the national health care insurance system. In accordance with the law passed on August 13, 2004, the attending physician is required to establish a health care plan for their patient and send a copy of this plan to the patient’s health insurance company to obtain agreement for reimbursement of 100% of the health care costs related to GD. Patient management is multidisciplinary and should be coordinated by a GD specialist, in conjunction with the Committee for the Evaluation and Treatment of Gaucher Disease (Comité d'Evaluation du Traitement de la maladie de Gaucher; CETG), the Reference Center for Lysosomal Diseases (Centre de Référence des Maladies Lysosomales; CRML) or a reference/competence center for inherited metabolic diseases (Centre de Référence/Compétence des Maladies Héréditaires du Métabolisme).

#### Specific medical treatments for GD

As the indication for treatment is not systematic, initial prescriptions must be validated, on a case-by-case basis and using a multidisciplinary approach, by a GD specialist belonging to the CETG.

In 2022, two-thirds of adult patients with GD being managed in France were receiving a GD-specific treatment. The indication for treatment is based on the presence of clinical, biological, and imaging criteria (see “Therapeutic management” section of the main article for details). Current treatments for GD generally lead to significant improvements in most clinical and biological disease characteristics within one to five years. GD-specific treatments can also greatly reduce the risk of bone and organ complications. In addition, early initiation of treatment may prevent some rare, but irreversible, late complications of GD such as fibrous splenomegaly, hepatic fibrosis, pulmonary fibrosis, pulmonary arterial hypertension (PAH), osteoarthritis secondary to osteonecrosis, and bone deformities after vertebral compression or fracture. ERT is recommended in case of pregnancy to prevent worsening of the disease. Given the lack of validated criteria for discontinuing treatment once it has been initiated, GD treatments should be continued for life.

Two ERT drugs are currently marketed in France (imiglucerase, CEREZYME® and velaglucerase alpha, VPRIV®), both of which act by replacing the deficient enzyme. ERT is administered by intravenous infusion every two weeks, either in an outpatient hospital setting or at home, depending on patient preference. In addition, two oral SRT drugs, which act by inhibiting glucosylceramide synthase, have been developed: a ceramide analog (eliglustat; CERDELGA®), and a less specific and less potent D-glucose analog (miglustat; ZAVESCA®). Both ERT drugs can be used as first-line treatments for GD1, whereas imiglucerase is the only drug with marketing authorization for the treatment of GD3. Eliglustat can be used as an alternative treatment for GD1 in adults. In 2025, eliglustat has been approved by the European Medicine Agency for paediatric patients with GD1 who are 6 years and older with a minimum body weight of 15 kg, who are stable on ERT. Miglustat is used as a second-line treatment for adult patients with GD1 who cannot take either ERT or eliglustat. There is no specific treatment for GD2.

For further information on the two disease-specific therapeutic strategies for the treatment of GD (ERT and SRT), see Appendix 5.

#### Other treatment options

- Splenectomy is prone to complications and is no longer recommended (except in specific cases).
- Bone marrow transplant may be discussed in exceptional cases of GD in children.
- All necessary symptomatic (analgesics, etc.), orthopedic, rehabilitation and disability management measures for patients should be undertaken.
- Therapeutic patient education programs can be offered.
- Health care professionals and patients should be informed of the existence of patient organizations through reference or competence centers, institutional websites and ORPHANET.

### Follow-up

Follow-up (see Appendix 4) should include a clinical examination, biological analyses to monitor disease biomarkers once or twice a year when the treatment objectives have been achieved, and imaging evaluations (MRI or abdominal ultrasound, bone MRI, bone densitometry, etc.) initially every year and then every 3 to 4 years for patients with stable disease in whom therapeutic objectives have been achieved. Between visits to a specialist, intercurrent pathologies can be managed by the attending physician (i.e. the general practitioner), in collaboration with the GD specialist.

## Main article

### Background

#### Definition of Gaucher disease

Gaucher disease (GD) is an autosomal recessive lysosomal disorder caused by a deficiency of glucocerebrosidase (also known as glucosylceramidase or β-acid glucosidase) or, in rare cases, of its activator, saposin C [1, 2]. Glucocerebrosidase hydrolyzes glucosylceramide (or glucocerebroside), a sphingolipid resulting from the degradation of cell membranes, into ceramide (or cerebroside) and glucose. In GD, the glucocerebrosidase deficiency leads to the accumulation of undegraded glucosylceramide, mainly in the lysosomes of macrophages. These cells then adopt a characteristic morphology and are often referred to as Gaucher cells [3].

Classically, there are three main clinical forms of GD:

- Gaucher disease type 1 (GD1; OMIM#230,800, ORPHA77259) is clinically very heterogeneous, ranging from forms that remain asymptomatic throughout life to severe forms with onset in childhood. GD1 may be associated with varying degrees of organomegaly, cytopenia and bone involvement.
- Gaucher disease type 2 (GD2; OMIM#230,900 and #608,013, ORPHA77260)) is a very rare form with very early onset and a poor prognosis.
- Gaucher disease type 3 (GD3; OMIM#23,100 and #231,005, ORPHA77261) is a rare form associated with progressive encephalopathy with varying severity and systemic manifestations similar to those found in GD1.

#### Epidemiology

The average annual incidence of GD is around 1/50,000 births. However, it varies greatly between populations, and can be as high as 1/1000 births in the Ashkenazi Jewish population. The prevalence in France is estimated at 1/130,000 [4]. In 2022, 521 individuals living with GD (446 adults and 75 children) were registered in the French Gaucher Disease Registry (Registre Français de la Maladie de Gaucher; RFMG).

#### Patient management

The initial management of the patient should be multidisciplinary and coordinated by a physician specializing in GD or inherited metabolic disorders.

Management frequently involves a range of health care professionals, including the attending physician, as well as pediatricians, internal medicine specialists, hematologists, neurologists, hepatologists, rheumatologists, biochemists, histopathologists, geneticists, radiologists, hospital pharmacists, cardiologists, orthopedic surgeons, gynecologists-obstetricians, physical medicine and rehabilitation physicians, pulmonologists, and other professionals depending on the clinical picture. Paramedical health care professionals (physiotherapists, nurses, etc.) also play a crucial role in patient care.

### Objectives of the national diagnostic and care protocol

The aim of this French National Diagnosis and Care Protocol (Protocole National de Diagnostic et de Soins; PNDS) is to provide health care professionals with guidance for the optimal management and care of patients with GD. GD is a rare genetic disorder that is recognized by the French health insurance system as a chronic health condition.

The PNDS is not intended to be exhaustive and does not replace the individual responsibilities of the physician toward the patient.

The 2022 update of the PNDS was carried out by the members of the Committee for the Evaluation and Treatment of Gaucher Disease (Comité d'Evaluation du Traitement de la maladie de Gaucher; CETG). It was initiated at the request of the Reference Center for Lysosomal Diseases (Centre de Référence des Maladies Lysosomales; CRML), and was conducted following the methodology proposed by the French National Authority for Health (Haute Autorité de Santé, HAS; methodology guide available on the HAS website [5]) in accordance with the recommendations of the French Network for Rare Inherited Metabolic Diseases (G2M), which was established in 2020. The coordinator set the objectives, developed the timetable, defined the working groups, and identified the editors and reviewers (see Appendix 6). Each chapter of the PNDS was updated in two stages: drafting by a group of editors under the responsibility of a steering committee made up of individuals with the required fields of expertise, followed by validation of the draft during plenary sessions. The text was then reviewed and edited by a group of reviewers, working independently of the editors. The final text was approved by all working groups, the steering committee, and the coordinator on April 20, 2022.

As experts in GD, most members of the CETG have links with the pharmaceutical industry (invitations to conferences, interventions as speakers, project grants, etc.). However, none of the members has an exclusive relationship with any one industrial body. No members of the pharmaceutical industry were involved in drafting or reviewing the PNDS.

This PNDS is the reflect of the French guidelines, and might not be applicable to other countries, mostly outside Europe and North America, with limited possibilities for medical evaluation and treatment. The recommendations should therefore be interpreted in the context of the available resources.

### Description of the disease

#### Gaucher disease type 1

GD1 is the most common form and accounts for 95% of cases. In France, the mean age of onset is 15 years and the mean age at diagnosis is 22 years [4]. The most common manifestations leading to the diagnosis of GD1 are thrombocytopenia, splenomegaly, and bone involvement, such as avascular osteonecrosis and bone infarcts.

Patients with GD1 can present with any of the following manifestations:

- Splenomegaly: a very frequent manifestation of GD1 (present in over 90% of cases), which can be severe and associated with significant pain in the event of splenic infarction.
- Hepatomegaly: occurs in 60–80% of cases.
- Cytopenia: including thrombocytopenia, anemia, and, more rarely, leukopenia.
- Bleeding anomalies: usually moderate and including epistaxis (sometimes present since childhood), bleeding gums, spontaneous bruising, petechiae, or a history of hemorrhage during childbirth, surgery or after trauma.
- Bone damage: associated with acute pain in the form of bone crises (occurring predominantly in the pelvis and lower limbs and, more rarely, in the upper limbs), and/or chronic bone and joint pain. The bone pain may be associated with infarcts, avascular osteonecrosis, or pathological fractures. Local and systemic signs of inflammation (fever and edema) may also be present, resulting in an “osteomyelitis-like” clinical picture. Osteopenia or osteoporosis may occur, even in young patients. Bone involvement may also be asymptomatic and only detected during imaging examinations [6].
- Asthenia: a frequent manifestation of GD that can have an impact on the school and socioprofessional lives of the patients as well as on overall quality of life (as assessed using the EQ-5D-5L instrument) [7].
- Other manifestations: rare manifestations are listed in Appendix 7.

##### Gaucher disease type 1 in children

The clinical presentation of GD1 in children is similar to that in adults. The early onset of symptoms generally correlates with the presence of more severe disease. Splenomegaly is found in about 95% of children with GD1. In most cases, if GD1 is left untreated, it leads to delayed growth and/or puberty [8, 9]. The bone manifestations in children with GD1 are also similar to those found in adults. Painful bone crises are common (30% of cases) [3]. Asthenia, pain, and quality of life can be assessed using age-appropriate instruments and scales.

#### Gaucher disease type 2

This form of GD is very rare, accounting for less than 1% of GD cases. Patients are usually asymptomatic at birth. However, GD2 usually manifests during the first year of life with progressive and severe neurological impairment, associated with irritability, hypertonia or hypotonia, epilepsy, and supranuclear palsy with strabismus that progresses to complete ophthalmoplegia. Hepatosplenomegaly is common. Ichthyosis may occur in patients with very early onset. The symptoms of GD2 usually progress to dysphagia, stridor, cachexia, and death within the first 3 years of life [10, 11]. There is no specific therapy for GD2 and therefore management involves symptomatic treatment and palliative care [12–14].

A rare perinatal–lethal form of GD2 has also been described. In these rare cases, the disease manifests during the antenatal period, resulting in death in utero or soon after birth, often following a premature delivery. Non-immune hydrops is often present, sometimes associated with antenatal thrombocytopenia, fetal immobility, ichthyosis, hepatosplenomegaly, and arthrogryposis [15–17].

#### Gaucher disease type 3

This rare form of GD accounts for 5% of GD cases in France. The clinical picture is very heterogeneous, and patients may be made in adulthood only. In addition to the systemic manifestations found in GD1, patients with GD3 also have neurological manifestations that may be subtle but often appear during childhood.

The neurological manifestations of GD3 can include:

- An eye movement disorder, which may be the only abnormality on neurological examination. It is frequently, but not consistently, associated with convergent strabismus. Three specific tests need to be performed to identify eye movement anomalies:
- the eye-tracking test, during which the patient is asked to follow a moving target with their eyes;
- assessment of the oculocephalic reflex to determine whether the patient’s eyes remain focused on a fixed point in front of them when they move their head horizontally and vertically;
- the saccades test, which involves monitoring the patient’s eye movements when they are asked to look rapidly to the left and right, or up and down.

As the origin of the eye movement disorder in GD3 is supranuclear, typically only the saccades are abnormal, and predominantly horizontal. These eye movements may be slow or even absent (gaze paralysis), causing a compensatory blink of the eyelids during examination. The results of the eye-tracking test and the oculocephalic reflex assessment are normal. The ocular saccades test should therefore be performed systematically, particularly as the eye movement abnormality is only rarely reported by patients as reason for consultation but maybe noted by the family.

- Motor disorders, including cerebellar syndrome, tremors, myoclonus (which may take the form of an irregular tremor), dystonia, parkinsonism, and pyramidal tract dysfunction, which may be associated with spasticity in severe cases.
- Epilepsy with various types of seizure occurring in the same patient, including generalized tonic–clonic seizures (with loss of consciousness) and generalized myoclonic seizures (without loss of consciousness).
- Cognitive and behavioral disorders, including intellectual development disorders, learning disorders, autism spectrum disorders, attention-deficit/hyperactivity disorders and cognitive decline in adults. Screening can be performed using the Mini-Mental State Examination and Frontal Assessment Battery at Bedside tests [18, 19].

The most severe form of GD3 is characterized by chronic encephalopathy, which progressively worsens and may lead to severe disability, and is associated with motor disorders (including myoclonus), cognitive disorders, and severe drug-resistant epilepsy (also referred to as progressive myoclonic epilepsy).

GD3 can also cause thoracic kyphosis, even in the absence of vertebral compression. Corneal opacity, valvular and ascending aorta calcification, and hydrocephalus may also occur in patients with homozygous p.D409H (c.1342G > C) variants [20].

### Complications

Disease progression in GD patients can be marked by:

- A hemorrhagic syndrome, associated with thrombocytopenia in 60 to 90% of cases, and/or with platelet and coagulation disorders [3]. Despite low platelet counts, these hemorrhagic syndromes are rarely severe.
- Bone damage can occur at any time during the disease course but is less common in treated patients [4]. Pathological fractures and avascular osteonecrosis, in particular of the femoral head and tibial plateau, can be complicated by osteoarthritis and may cause chronic pain. Due to the impact of these bone manifestations on the functional prognosis, joint replacement surgery may be indicated in some cases [3]. More rarely, vertebral compression may lead to thoracic kyphosis or a gibbus deformity. Bone complications can have a significant impact on patient quality of life and daily functioning [21].
- An increased risk of hematological malignancies, gammopathies, and solid tumors, including monoclonal gammopathy of undetermined significance, myeloma and lymphoma, and hepatocellular and renal carcinomas [22–27].
- An increased risk of Parkinsonian disorders [28, 29].
- An increased risk of peripheral neuropathy in GD1 [30].

Patients who have undergone splenectomy may have a more severe disease course [31], due to an increased risk of bone events [32], liver fibrosis or even cirrhosis [33, 34], and PAH [35].

### Diagnosis

In the absence of evidence of any other etiology after carrying out a complete clinical examination, a diagnosis of GD should be considered in a range of clinical contexts (Appendix 8) and glucocerebrosidase activity should be measured. Diagnosis of GD is based on demonstration of reduced or absent glucocerebrosidase activity. In case of glucocerebrosidase deficiency, molecular analysis of the *GBA1* gene should be performed to confirm the diagnosis and determine the genotype. The diagnostic tree is shown in Appendix 9.

In patients with suspected GD with normal glucocerebrosidase activity and elevated plasma biomarkers, diagnostic tests to confirm the presence of the exceptionally rare form of GD caused by deficiency of the glucocerebrosidase activator, saposin C, should be considered [36].

The test to measure glucocerebrosidase activity should be carried out by a reference biomedical laboratory (see Appendix 1). Traditionally this test is performed on leukocytes, but it is increasingly being carried out on dried blood spots as part of a multiplex assay [37]. This multiplex assay can be used to diagnose several lysosomal and peroxisomal disorders, such as Niemann-Pick A/B disease (acid sphingomyelinase deficiency), which may have a clinical presentation very similar to that of GD.

Expertise is essential for correct interpretation of the test results, particularly for tests performed on dried blood spots. In case of abnormal or inconclusive results, further tests should be performed (by a reference biomedical laboratory) before a diagnosis of GD is made.

A bone marrow aspirate is not recommended for diagnosing GD. If performed (i.e., to investigate thrombocytopenia, splenomegaly, monoclonal spikes, etc.), it may reveal the presence of characteristic macrophages (known as Gaucher cells); however, the absence of these cells is inconclusive as they are sometimes difficult to detect or identify.

### Initial assessment

The initial assessment should include a full clinical examination (with dental and ophthalmological assessment), biological analyses and imaging evaluations (see Appendix 4, Table 1).

**Table 1:** Diagnosis and initial assessment of Gaucher disease (in children and adults)

Clinical Examination
Measurement of glucocerebrosidase activity (to help establish the diagnosis^a^)
Genotyping of the *GBA1* gene
Cytochrome P450 2D6 genotyping (prior to prescribing eliglustat): unnecessary in cases of GD2 and GD3 due to absence of marketing authorization
Complete blood count
Hemostasis assessment^b^
Biochemical assessment^c^
Serum protein electrophoresis and in case of abnormality immunofixation^d^: non-systematic in children
Biomarker testing^e^
Abdominal MRI^f^ or ultrasound
Bone MRI^f^ (spine, femurs, pelvis, tibiae, or whole-body)
Bone X-rays (full skeletal)
Chest X-ray
Bone densitometry
Electrocardiogram
Echocardiography
Chest HRCT scan + RFT: systematic in children and indicated in adults with clinical signs of lung involvement
Neuropsychological and quality of life assessment: systematic in children

ERT: Enzyme Replacement Therapy, *GBA1:* Glucocerebrosidase Gene*,* GD: Gaucher disease, HRCT: High Resolution Computed Tomography, MRI: Magnetic Resonance Imaging, RFT: Respiratory function test

^a^In very rare cases, when there is a strong suspicion of GD but normal glucocerebrosidase activity, patients should be assessed for saposin C deficiency

^b^Prothrombin time (PT) and activated partial thromboplastin time (APTT)

^c^Ionogram, and analyses of the glucose, creatinine, urea, phosphocalcic (phosphorus, calcium, vitamin D), and liver (alkaline phosphatase, ALP; aspartate aminotransferase, AST; alanine aminotransferase ALT, gamma-glutamyl transferase, GGT; and bilirubin) profiles

^d^A bone marrow aspirate should also be performed in case of a monoclonal spike

^e^Chitotriosidase, CCL18, and LysoGL1

^f^Procedure to perform MRI in GD: https://cetl.net/maladies-lysosomales/cetg-maladie-de-gaucher/documents-d-aide-a-la-prise-en/documents-d-information-pour-les/article/procedure-pour-la-realisaiton-des

Depending on the clinical presentation, further investigations should be considered.

#### Biological analyses

- Complete blood count to assess the severity of cytopenia. The blood count may be normal in patients who have undergone splenectomy.
- Ferritin levels are frequently high, but transferrin saturation is normal.
- Serum protein electrophoresis, and in case of abnormality, immunofixation [38].
- Prothrombin time and activated partial thromboplastin time. If abnormalities are detected, further tests should be conducted to check for the presence of one of the coagulation factor deficiencies sometimes found in patients with GD [39, 40].
- Liver function tests, including measurement of the activity of the aspartate and alanine transferases, gamma-glutamyl transferase and alkaline phosphatase, and assessment of total bilirubin.
- Creatinine and glomerular filtration rate assessments.
- Blood ionogram, blood glucose and blood albumin levels.
- Calcium, phosphorus, and 25-hydroxy vitamin D concentrations. Vitamin D deficiency is more common in patients with GD than in the general population [41].
- Folic acid and vitamin B12 levels.
- Lipid profile test. High-density lipoprotein cholesterol levels are often low in patients with GD [42].

#### Biomarkers

Measurement of the following three plasma biomarkers for GD is recommended: glucosylsphingosine (LysoGL1), as a marker of sphingolipid (glucosylceramide) accumulation; and chitotriosidase and C–C motif chemokine ligand 18 (CCL18) as markers of macrophage activation.

### Glucosylsphingosine (LysoGL1)

The levels of this biomarker are always high in patients with GD, due to deficiencies in glucocerebrosidase activity or saposin C [36]. LysoGL1 is more sensitive and specific than other GD biomarkers [43]. Although there is great variability in LysoGL1 plasma levels between patients at the time of diagnosis [44], higher levels of this marker correlate with increased severity of the hematological manifestations of GD (hepatosplenomegaly and cytopenia), but not with bone damage [36, 45, 46]. Plasma LysoGL1 levels can be analyzed using a multiplex assay, allowing the simultaneous measurement of the levels of other lysosphingolipids and differential diagnosis of Niemann-Pick disease types A/B and C [47]. LysoGL1 can also be measured on dried blood spots, however, this method was not being used in France in 2022.

### Chitotriosidase

Chitotriosidase activity is usually significantly elevated in patients with GD, whereas more moderate increases may be observed in other lysosomal and non-lysosomal pathologies [48]. However, around 6% of the general population have been found to have severe chitotriosidase deficiency, due to the presence of a homozygous 24-base pair duplication in the chitotriosidase gene *CHIT1* [49]. This polymorphism has no clinical impact but prevents the use of chitotriosidase as a biomarker for GD in patients who are homozygous for this variant.

### C–C motif chemokine ligand 18 (CCL18)

CCL18 levels are elevated in patients with GD. As the sensitivity and specificity of this biomarker are comparable to those of chitotriosidase, it can be used as an alternative in cases of chitotriosidase deficiency [50].

#### Sequencing of the GBA1 gene

*GBA1* gene analysis should be performed in all patients to confirm the biochemical diagnosis. In case of suspected saposin C deficiency (exceptional), sequencing of the prosaposin (*PSAP*) gene should be performed. Over 500 pathogenic variants in the *GBA1* gene have been described. The main genotype/phenotype correlations are:

- c.1226A > G (N370S, renamed as p.Asn409Ser): the presence of this variant in the homozygous or heterozygous state excludes the diagnosis of GD2 and GD3;
- c.1448 T > C (L444P, renamed as p.Asp483Pro): this variant is generally associated with GD2 or GD3 if present in the homozygous state;
- c.1342G > C (D409H renamed as p.Asp448His): this variant is present in the homozygous state and is associated with the presence of valvular calcification in GD3.

Patients carrying two "null" variants resulting in a complete lack of glucocerebrosidase activity (RecNciI, c.84dup, etc.) do not survive beyond the perinatal period (perinatal–lethal form of GD2). Intra-familial phenotypic variability are frequent, suggesting the influence of modifier genes, although the mechanism remains poorly understood.

#### Imaging evaluations

The following imaging evaluations should be performed:

- Abdominal MRI or ultrasound
- Bone MRI can be used to:
- Quantify and monitor the degree of bone infiltration by Gaucher cells, which manifests as abnormal low signal intensity on T1-weighted and T2-weighted image sequences;
- assess the extent of bone damage and determine whether the lesions are recent or older (oedema indicates recent bone infarct or osteonecrosis)
- specify certain lesions revealed on standard X-rays.

Several scoring systems have also been developed to allow semi-quantitative analyses of bone infiltration; however, although these scores are used for clinical research in reference centers [51], they are not routinely performed in clinical practice. Whole-body MRI is available in some centers and enables abdominal MRI and bone MRI to be performed at the same time. Regardless of the method used, the radiologist must be able to quantify the level of bone infiltration. If a bone MRI is unavailable or is contraindicated, a Technetium-99m bone scan can be performed.

- Skeletal X-rays, which are not routinely performed but can be useful in certain cases to detect Erlenmeyer flask deformity, osteosclerosis following bone infarcts, degenerative arthropathies following osteonecrosis, cortical thinning, lytic lesions, fractures, and for the follow-up of prosthetic replacements and osteosynthesis interventions.
- Chest X-rays
- Bone densitometry of the lumbar spine and femoral neck. Results should be interpreted as follows:
- osteopenia: a Z-score < -2 in adult patients under the age of 50 years and premenopausal women, or a T-score between -1 and -2.5 in patients aged 50 years or over or in postmenopausal women;
- osteoporosis: a Z-score ≤ -3 in adult patients under the age of 50 years and in premenopausal women, or a T-score ≤ -2.5 in patients aged 50 years or over or in postmenopausal women.

For GD2 and GD3, other examinations should be carried out, depending on the clinical picture (see Appendix 4, Table 1):

- Eye movement assessments, if possible, by electrooculography to look for saccadic abnormalities, which may not by visible during the clinical examination.
- Ophthalmological examination (ophthalmoscopy).
- Auditory brainstem evoked responses in case of auditory or language disorders.
- MRI of the brain to detect basal ganglia and white matter abnormalities or hydrocephalus in case of neurological disorders other than saccades.
- Electroencephalogram in case of epilepsy.
- Neuropsychological assessments.
- Cardiac ultrasound to check for heart calcification, PAH or cardiomyopathy.
- Chest X-ray or high-resolution computed tomography scan to check for the presence of interstitial lung disease.
- Respiratory function tests in case of shortness of breath or interstitial lung disease.

#### Delivering the diagnosis and disease information to the patient

During the consultation, patients (or their parent or legal guardian) should be provided with information about GD and its clinical forms, as well as information about follow-up and the different treatment options (see Appendix 10). Genetic counseling, along with psychological and social care support, should also be proposed. A request for consent for genotyping should be made during this consultation. The patient (or their parent or legal guardian) should also be informed of the existence of national and international patient associations and the possibility of being included in the RFMG (see Appendix 2), the French national rare disease database (Banque Nationale de Données Maladies Rares) and the ELODIE-MG national collection of GD biological samples (see Appendix 3). Patients (or their parent or legal guardian) should also receive an emergency card, issued by the Ministry of Health (see Appendix 11). The attending physicians should also be informed of the diagnosis: they are a key partner in patient management.

#### Genetic counseling and prenatal/pre-implantation diagnosis

Once the diagnosis of GD has been made, it is recommended that the patient be referred for genetic counseling.

For the family of a patient with GD, genetic counseling will aim to:

- Diagnose any potential cases of GD in the patient's siblings, allowing them to access appropriate treatment and monitoring.
- Discuss the value of testing adult relatives (parents, siblings, and offspring) to determine if they are heterozygous carriers of the disease-causing variant. Screening of the patient’s partner or spouse is especially useful in cases of consanguinity or if the spouse is of Ashkenazi Jewish origin.

Prenatal/pre-implantation diagnosis may be offered to couples at risk of having a child with severe GD (perinatal–lethal form of GD2 or GD3).

## Therapeutic management

### Treatment initiation criteria

The initiation of a specific treatment for GD is not systematic. In 2022, two-thirds of adult patients being followed up in France were receiving GD-specific treatment. Ideally, the indication for treatment should be validated by experts from a reference center during a multidisciplinary consultation meeting held by the CETG. The decision is then shared with the patient.

Specific treatments for GD are indicated if one or more of the following criteria are met:

- Symptomatic thrombocytopenia and/or platelet count < 50 × 10^9^/L

The CETG recommends initiating GD treatment in patients with symptomatic thrombocytopenia and in those with platelet counts below 50x10^9^/L. In the absence of any bleeding anomalies, treatment is not recommended for patients with platelet counts above 100x10^9^/L. For patients with platelet counts between 50 and 100x10^9^/L, treatment should be considered on a case-by-case basis during the multidisciplinary consultation meetings.

- Symptomatic anemia and/or a hemoglobin level ≤ 10 g/dL
- Severe and/or painful splenomegaly
- Past or present bone damage, associated with:
- painful bone crises, avascular osteonecrosis, bone infarcts, or pathological or bone fragility fractures;
- lytic lesions or cortical thinning.
- Osteoporosis (T-score ≤ -2.5 for patients aged 50 years or over, or a Z-score ≤ -3 for those aged under 50 years).
- Other visceral manifestations related to GD: interstitial lung disease, liver fibrosis, cardiac involvement, etc.
- GD3

There is no specific treatment for GD2.

In the absence of any formal criteria for treatment initiation, the indication for treatment should be discussed during a multidisciplinary consensus meeting, on a case-by-case basis, particularly for patients in which GD is associated with impaired quality of life and in all cases involving symptomatic children.

#### GD-specific treatments

Once started, GD-specific treatment is usually continued for life (see Appendix 5).

Treatment discontinuation usually results in the reoccurrence of clinical signs, preceded by an increase in the levels of biomarkers [52]. There are currently two therapeutic strategies for the treatment of GD: enzyme replacement therapy (ERT) and substrate reduction therapy (SRT).

### Enzyme replacement therapy (ERT)

The aim of ERT is to compensate for the deficiency in glucocerebrosidase activity. In 2022, two ERT drugs were being marketed in France:

- Imiglucerase (CEREZYME.®), which obtained marketing authorization in November 1997 [53]
- Velaglucerase alpha (VPRIV.®), which obtained marketing authorization in August 2010 [54]

### Substrate reduction treatment (SRT)

The principle of SRT is to inhibit the activity of glucosylceramide synthase, the enzyme which catalyzes the synthesis of glucosylceramide from glucose and ceramide. In 2022, two SRT drugs were being marketed in France:

- Miglustat (ZAVESCA.®), which obtained marketing authorization in Europe in November 2002, or generics [55]
- Eliglustat (CERDELGA®), which obtained marketing authorization in Europe in January 2015 [56]. In 2025, eliglustat has been approved by the European Medicine Agency for paediatric patients with GD1 who are 6 years and older with a minimum body weight of 15 kg, who are stable on ERT.

Miglustat, an imino-sugar and glucose analog, acts as a competitive inhibitor of glucosylceramide synthase.

Eliglustat is a ceramide analog that functions as a more specific and potent glucosylceramide synthase inhibitor. Eliglustat is metabolized by cytochrome P450 (CYP2D6). Prescription of the drug and the recommended dosage depend on the CYP2D6 metabolism profile of the patient, which can be established by a saliva or blood test (see Appendix 5).

#### Therapeutic strategy criteria

ERT and eliglustat are first-line treatments [57–65]. In case of severe disease, the CETG recommends ERT.

Miglustat is a second-line treatment for adult GD1 patients who cannot be given ERT or eliglustat.

Switching between therapeutic classes may be considered in adult patients with GD1 with poorly controlled disease despite treatment; in case of adverse events, difficult-to-manage drug interactions, or comorbidities; or according to the preferences of the patient [66–71].

#### Anticipated therapeutic effects

Patients with GD1 initiating ERT or eliglustat treatment show rapid improvements in clinical and biological disease characteristics during the first year, followed by more gradual improvements during subsequent years. The therapeutic aims are as follows:

- Resolution of anemia (usually achieved after 12 to 24 months of treatment).
- Resolution of thrombocytopenia (platelet counts ≥ 100 × 10^9^/L, which is the bleeding risk threshold). Moderate thrombocytopenia persists in some patients despite treatment [72–78].
- Reduction and stabilization of liver and spleen size (a return to normal size is not always possible).
- Reduction in bone pain and prevention of clinical and radiological bone events. Improvements in bone infiltration, as assessed by MRI, can usually be achieved within the first 24 months of treatment [79].
- Stabilization or an increase in bone mineral density. Bone mineral density has been shown to improve after 24 months of treatment and may normalize after 8 years of treatment [73–78].
- Prevention or reduction of interstitial lung disease and PAH, although these manifestations are generally unresponsive to GD treatments.
- Resolution of asthenia, normalization of the patient’s school and socio-professional life. An improvement or normalization of quality of life is also frequently observed.
- Prevention or correction of delayed growth and/or puberty.

ERT is not effective in patients with GD2 [12, 13]. For this form of the disease, management involves symptomatic and palliative care [14].

The efficacy of ERT for the treatment of the various types of cytopenia, organomegaly and bone involvement in patients with GD3 is comparable to that observed for patients with GD1 [80–82]. In contrast, ERT has no effect on the evolution of thoracic kyphosis, or on the pulmonary and neurological manifestations associated with GD3, apart from, at best, stabilization of the ophthalmoplegia [80, 83, 84].

Levels of GD biomarkers decrease significantly (by about 50%) during the first year of treatment, and then decline more slowly thereafter [36, 85, 86]. The decreases in biomarker levels observed for ERT and eliglustat appear to be quite similar [87]. The absence of a decrease in biomarker levels, or subsequent increase in the levels of these biological parameters should be seen as a warning sign of a possible insufficiency or a decline in treatment effectiveness.

#### Non-specific treatments

##### Splenectomy

In theory, as specific treatments are available, there is no longer an indication for splenectomy in GD, except in case of splenic rupture or in patients who fail to respond to GD-specific treatments and have severe and persistent thrombocytopenia. Such cases of persistent thrombocytopenia despite treatment are exceptional and are usually associated with severe nodular and fibrous splenomegaly [88]. If a decision is made to perform a splenectomy, all vaccinations (as recommended by the French High Council for Public Health (Haut Conseil de la Santé Publique [89]) must be given at least two weeks before the surgery takes place. Patients who have undergone a splenectomy should be made aware of the risk of infection by encapsulated bacteria. They must also be provided with a splenectomy card to inform health care professionals of their status.

##### Bone marrow transplant

Bone marrow transplants are not indicated for GD1 in France because of the higher benefit/risk ratios associated with GD-specific treatments. They can be offered on a case-by-case basis for GD3.

### Non-specific medical treatments

- Painkillers

The treatment of chronic bone pain and bone pain crises may require level III analgesics (strong opioids).

- Vitamin D

The aim is to maintain normal levels (≥ 75 nmol/L or 30 ng/mL) of calcidiol (25-hydroxyvitamin D). Vitamin D supplements can be provided in the form of cholecalciferol (vitamin D_3_, human form) or ergocalciferol (vitamin D_2_, plant form) [41]. For children, the usual vitamin D supplement recommendations should be followed [90].

- Calcium

An adequate intake of between 1 g and 1.5 g per day must be ensured.

- Oral bisphosphonates

These treatments are only indicated for adults with vertebral compression associated with osteoporosis. No data are available regarding the effectiveness of this treatment for reducing the risk of fracture. Oral bisphosphonates are not indicated for women of childbearing age.

- Anti-epileptics: in case of epilepsy.
- Vaccinations

Except for patients who have undergone a splenectomy (see above), there are no specific vaccination recommendations for GD. For general guidance, including recommendations for SARS-CoV-2 vaccination, refer to the vaccination calendar of the French Ministry of Health and Solidarity (Ministère de la Santé et de l’accès aux soins) [91].

- Treatment with antibiotics

Antibiotic treatment may be required in the following situations:

- antibiotic prophylaxis before surgery for joint replacement, in accordance with the recommendations of the French Society of Anesthesia and Intensive care Medicine (Société Française d'Anesthésie et de Réanimation) [92];
- preventive antibiotic treatment in patients undergoing splenectomy (see above);
- curative antibiotic treatment for osteomyelitis (exceptional): these infections may occur after a biopsy of a bone infarct. Therefore, it should be noted that bone biopsy must absolutely be contraindicated in this context;
- curative antibiotic treatment, targeting encapsulated bacteria when there is intercurrent infection in a patient after a splenectomy.

### Other non-specific treatments

- Orthopedic treatment
- crutches in case of avascular osteonecrosis in the lower limbs;
- a spinal brace in case of vertebral compression;
- osteosynthesis for fractures, joint replacement surgery, and arthrodesis, as recommended by the orthopedic surgeon.
- Physical interventions, rehabilitation, and physiotherapy

Treatment must be adapted depending on the functional abilities of the patient and take into account any disabilities that remain post-orthopedic treatment.

- Interventions to improve or correct auditory, ocular and orthoptic impairment (rehabilitation of eye movement disorders). The correction of sensory disabilities mainly concerns patients with GD3.
- Dental and oral care.

Rigorous dental hygiene should be ensured, due to the frequency of caries and dental fragility [93]. Specialist care is essential for cases of mandibular bone damage.

- Managing multiple disabilities

In cases when disease progression leads to multiple disabilities, mainly in patients with neurological manifestations, it may be necessary to recommend adjustments to improve day-to-day life (home adaptations, accessible vehicles, etc.) and to prescribe medical devices: crutches, spinal braces, adapted seats, day and/or night splints, orthopedic shoes, walking frames, manual or electric wheelchairs, anti-bedsore mattresses, medical beds, oxygen at home, suction equipment, tracheotomy tubes, implantable catheters, peripheral or central venous infusion kits, and nasogastric or gastrostomy tubes. Assistance should be provided by appropriate establishments (e.g. the departmental offices for persons with disabilities, specialist centers).

- Therapeutic education and information about patient rights and patient associations: see Appendix 10.

#### Therapeutic perspectives

- Treatment with a pharmacological molecular chaperone (ambroxol or arimoclomol) which aims to partially restore residual enzyme activity is undergoing clinical evaluation [94].
- The therapeutic challenge is to identify specific treatments that are disseminated to all affected tissues, particularly to zones that cannot be reached by current treatments, such as the central nervous system in GD3. For example, venglustat, a glucosylceramide synthase inhibitor that can cross the blood–brain barrier, is undergoing clinical evaluation in patients with GD3 [95, 96].
- Gene therapy is currently being evaluated as a strategy for replacing the deficient gene, either by transduction using a vector (in vivo gene modification) or by reinjection of genetically modified stem cells ex vivo. Clinical evaluation is ongoing [97–100].

### Specific situations

#### Pregnancy

Pregnancy and childbirth warrant a multidisciplinary approach involving rheumatologists, pediatricians, internal medicine specialists, hematologists, obstetricians, anesthetists, and hemostasis specialists. Indeed, in patients with severe or untreated disease, pregnancy can lead to worsening of GD symptoms, and GD may affect pregnancy and childbirth. Complications occurring during pregnancy and childbirth can include:

- Aggravation of thrombocytopenia and hemostasis disorders, which may lead to the contraindication of epidural anesthesia and cause post-partum hemorrhage. It is imperative to carry out a full hematological assessment, particularly a complete evaluation of the patient’s coagulation profile, prior to delivery.
- Painful bone crises (due to bone infarction or avascular osteonecrosis), osteopenia, and risk of pathological fractures.

The benefit/risk ratio of ERT during pregnancy is very favorable. Continuation of ERT during pregnancy therefore is advised. Initiating ERT should be considered in all pregnant women with GD. In addition, ERT reduces the risk of post-partum hemorrhage [101].

Animal studies have demonstrated that ERT has no direct or indirect harmful effects on gestation, embryonic or fetal development, childbirth, or post-natal development [101–104].

Given the lack of adequate data, eliglustat and miglustat are contraindicated during pregnancy [105]. Both treatments require women of childbearing age to use contraception. For patients initiating GD treatment who are planning to become pregnant, ERT should be preferred over SRT. Patients already receiving SRT who are planning a pregnancy should switch to ERT.

Men prescribed miglustat are required to use male contraceptives as this treatment alters spermatogenesis. The use of contraception should be continued for up to three months after stopping miglustat.

#### Breastfeeding

No specific data are available for breastfeeding women. However, it is likely that the replacement enzymes used in ERT are destroyed by the child's gastrointestinal tract. ERT can be continued when breastfeeding. Eliglustat and miglustat are contraindicated in breastfeeding women.

#### Cancer and blood disorders

Treatment for cancer and blood disorders should follow the usual recommendations. There are no published data indicating that changes to treatment should be recommended. If the patient is not being treated for GD, initiation of ERT can be considered to minimize the cumulative impact of chemotherapy and GD on disease manifestations such as cytopenia, organomegaly and osteopenia; however, the therapeutic benefit may by limited due to time required for ERT to result in improvements in these disease characteristics. Prescribing eliglustat requires vigilance due to the risk of drug interactions [22, 106].

#### Parkinsonian disorders

There is a higher risk of developing Parkinson's Disease (PD) in GD patients compared to the general population. In some patients, the diagnosis of GD is made at the same time as they begin management for PD. GD-specific treatments have no impact on the disease course of patients with established PD [107–109].

### Monitoring Gaucher disease

#### The purpose of monitoring

Monitoring of GD (Appendix 4) is essential in order to:

- Assess the severity of the disease, detect possible complications, and identify comorbidities.
- Assess responses to treatment: tolerance, compliance, dosage, and administration (home-based treatment, foreign travel, pregnancy planning, etc.).
- Re-evaluating whether to refrain from treating untreated patients (e.g. in patients planning a pregnancy or undergoing surgery).
- Evaluate the psychological, familial, and socio-professional consequences of the disease.

Patient monitoring should include regular clinical examinations and biological analyses, and systematic imaging evaluations [73, 110–113].

#### Monitoring details and frequency

Schedules for monitoring GD patients, including the details of the necessary clinical examinations, biological analyses, and imaging evaluations are laid out in detail in Tables 2 and 3 and in Appendix 4.

**Table 2:** Schedule for GD1 and GD3 follow-up examinations

Examinations	1st Year		2nd Year^f^	3rd Year^f^	Following years^g^
	M6^f^	M12			
Clinical examination	X	X	Six-monthly	X^h^	X^h^
Complete blood count	X	X	Six-monthly	Six-monthly	Six-monthly
Hemostasis assessment^a^		X	X^h^	X^h^	X^h^
Biochemical assessment^b^	X	X	Six-monthly	Six-monthly	Six-monthly
Serum protein electrophoresis^c^		X	X	X	X
Biomarker testing^d^	X	X	Six-monthly	Six-monthly	X^h^
Abdominal MRI^e^ or ultrasound	X	X	Six-monthly	X	Every 3 years^i^
Bone MRI^e^ (spine, femurs, pelvis, tibiae, or whole-body)		X		X	Every 3–4 years^i^
Bone densitometry			X		Every 3–4 years^i^
Echocardiography, Chest HRCT scan, RFT	As required, according to clinical signs				
Testing for ERT antibodies	As required in case of an allergic reaction or reduced effectiveness in patients treated with ERT				

ERT: Enzyme Replacement Therapy, HRCT: High Resolution Computed Tomography, M: month, MRI: Magnetic Resonance Imaging, RFT: Respiratory function test

^a^Prothrombin time (PT) and activated partial thromboplastin time (APTT)

^b^Ionogram, and analyses if the glucose, creatinine, urea, phosphocalcic (phosphorus, calcium, and vitamin D), and liver (alkaline phosphatase, ALP; aspartate aminotransferase, AST; alanine aminotransferase ALT, gamma-glutamyl transferase, GGT; and bilirubin) profiles

^c^Complete with immunofixation, the plasma immunoglobulin-free light chain assay and a bone marrow aspirate in case of a monoclonal spike. In case of Monoclonal Gammopathy of Undetermined Significance (MGUS), parameters should be monitored every 2 years in patients under the age of 50 years, and every year in those over the age of 50 years

^d^Chitotriosidase, CCL18, and LysoGL1

^e^Procedure to perform MRI in GD: https://cetl.net/maladies-lysosomales/cetg-maladie-de-gaucher/documents-d-aide-a-la-prise-en/documents-d-information-pour-les/article/procedure-pour-la-realisaiton-des

^f^A follow-up at M3 can be added. From the second year onwards, clinical and biological monitoring should be performed annually, except for the blood count, which must be monitored every six months

^g^This schedule is for patients with stable disease in whom therapeutic objectives have been achieved. The schedule can be modified depending on the disease course

^*h*^Six-monthly in children

^i^Every two years in children

**Table 3:** Specific neurological follow-up of GD3

Examinations	Initial Report	1st Year				2nd Year	Subsequent years
		M3	M6	M9	M12		
Neurological consultation	X	X	X	X	X	Six-monthly	Six-monthly
Eye movement examinations	X		X		X	Six-monthly	Six-monthly
Ophthalmic examination	X				X	X	X
Neuropsychological assessment	X						Assessment at age 3, 6, 12 and 18 years and as required in case of clinical symptoms (educational issues, etc.)

M: month

## Data Availability

Data sharing is not applicable to this article as no datasets were generated or analyzed during the update of this PNDS.

## Appendix 1: French reference biomedical laboratories

**Table:**

Name and contact details of the laboratory manager	Tests carried out	Link to the test catalog
**Prof. Soumeya BEKRI** **Laboratory of Metabolic Biochemistry** Rouen University Hospital 1, rue de Germont, 76,000 Rouen, France Tel: 00 33 2 32 88 01 25 / 81 24 Fax: 0033 2 32 88 83 41 E-mail: soumeya.bekri@chr-rouen.fr	Glucocerebrosidase Chitotriosidase LysoGL1 *GBA1 genotyping, PSAP* Common polymorphism of the *CHIT1* gene	https://chu-rouen.manuelprelevement.fr/ResultNew.aspx
**Prof. Marc BERGER** **Department of Biological Hematology** Center for Biological Resources (CBR) Auvergne Estaing University Hospital 1, place Lucie et Raymond Aubrac, 63,003 Clermont-Ferrand Cedex 1, France Tel: 0033 4 73 75 03 68 Fax: 0033 4 73 75 02 15 E-mail: mberger@chu-clermontferrand.fr	Glucocerebrosidase (by flow cytometry) CCL18 CRB Auvergne: The GD national biological collection ELODIE-MG	https://chu-clermontferrand.manuelprelevement.fr/DetailNew.aspx?id=A13696
**Dr Catherine CAILLAUD, Dr Edouard LE GUILLOU** **Laboratory of Metabolic Biochemistry** Necker Hospital—Sick Children, Tour Lavoisier (4th floor) 149, rue de Sèvres, 75,015 Paris, France Tel: 0033 1 44 49 58 58 Fax: 0033 1 44 49 51 30 E-mail: catherine.caillaud@aphp.fr edouard.leguillou@aphp.fr	Glucocerebrosidase Chitotriosidase *GBA1* Genotyping, *PSAP* Common polymorphism of the *CHIT1* gene	https://nck.manuelprelevement.fr/
**Dr Roseline FROISSART, Dr Magali PETTAZZONI** **Laboratory of Biochemistry and Molecular Biology** UM Inherited Metabolic and Red Blood Cell Diseases Hospices Civils de Lyon Biology Center East, Hospital Cluster East 59, Boulevard Pinel, 69,677 Bron Cedex, France Tel: 0033 4 72 12 96 32 Fax: 0033 4 72 12 97 20 E-mail: roseline.froissart@chu-lyon.fr magali.pettazzoni@chu-lyon.fr	Glucocerebrosidase Chitotriosidase LysoGL1 *GBA1* Genotyping, *PSAP*	https://biobook.chu-lyon.fr/Home
**Dr Roselyne GARNOTEL** **Pediatric Research and Biology Department** 45, rue Cognacq Jay, 51,092 Reims Cedex, France Tel: 0033 3 26 78 79 55 Fax: 0033 3 26 78 84 56 E-mail: rgarnotel@chu-reims.fr	Glucocerebrosidase	http://guide-laboratoire.chu-reims.fr/
**Prof. Rosa-Maria GUEANT-RODRIGUEZ** **Laboratory of Biochemistry, Molecular Biology, Nutrition and Metabolism** Nancy University Hospital Allée du Morvan, 54,511 Vandoeuvre les Nancy, France Tel: 0033 3 83 15 34 55 Fax: 0033 3 83 15 37 85 E-mail: rm.rodriguez@chu-nancy.fr	Glucocerebrosidase Chitotriosidase	https://chu-nancy.manuelprelevement.fr/default.aspx
**Dr Foudil LAMARI** **Laboratory of Metabolic Biochemistry** Pitié Salpêtrière Hospital 47–83, Boulevard de l'Hôpital, 75,013 Paris, France Tel: 0033 1 42 16 21 90 E-mail: foudil.lamari@aphp.fr	Glucocerebrosidase LysoGL1	https://www.aphp.fr/professionnel-de-sante/adressez-un-prelevement-de-biologie/biologie-medicale-des-hopitaux
**Prof. Thierry LEVADE** **Laboratory of Metabolic Biochemistry** Toulouse University Hospital, Federal Biology Institute, TSA 40031 330, Avenue de Grande-Bretagne, 31,059 Toulouse Cedex 9, France Tel: 0033 5 67 69 04 81 or 0033 6 14 14 72 61 Fax: 0033 5 67 69 03 77 E-mail: levade.t@chu-toulouse.fr	Glucocerebrosidase Chitotriosidase	https://chu-toulouse.manuelprelevement.fr/default.aspx
**Prof. Marie-Anne LORIOT** **Medical Biology Laboratory, Biochemistry Department** Hôpital Européen Georges Pompidou 20, rue Leblanc, 75,908 Paris Cedex 15, France Tel: 0033 1 56 09 38 82 or 39 01 Fax: 0033 1 56 33 93	Cytochrome *CYP2D6* genotyping	https://aphp.manuelprelevement.fr/ (blood sample)
**Dr Sabrina VERGNAUD** **Rare Enzyme Inherited Diseases—CGD** SB2TE—IBP Grenoble Alpes University Hospital, CS 10217, 38,043 Grenoble Cedex 9, France Tel: 0033 4 76 76 54 83 or 59 05 Fax: 0033 4 76 76 56 08 E-mail: svergnaud@chu-grenoble.fr	Glucocerebrosidase Chitotriosidase	http://biologie.chu-grenoble.fr/
**Prof. Céline VERSTUYFT** **Molecular Genetics and Pharmacogenetics Laboratory** Bicêtre Hospital 78, rue du général Leclerc, 94,275 Le Kremlin Bicêtre, France Tel: 0033 1 45 21 35 87 or 35 88 Fax: 0033 1 45 21 35 91	Cytochrome *CYP2D6* genotyping	https://aphp.manuelprelevement.fr/ (saliva sample or blood sample)

## Appendix 2: The French GD registry (RFMG)

The RFMG was initiated by the CRML in response to the requirements of the first National Plan for Rare Diseases (PNMR 2005–2008) to centralize data on rare diseases.

It was accredited by the Registries Committee (InVS/Inserm) in 2009. It is coordinated by Dr N. Belmatoug and Dr. Y. Nguyen. The Scientific Directors are Dr J. Stirnemann and Dr. Y. Nguyen. The registry was designed and developed by D. Hamroun (PhD), a database, registry, and cohort coordinator. The CETG, which is chaired by Dr F. Camou, is an official partner.

The registry is supported by the French Patient Association for Lysosomal Diseases (Vaincre les Maladies Lysosomales; VML). Information is collected by the clinical research associates of the CRML (Karima Yousfi). An annual report is also produced (Dr J. Stirnemann and Dr Y. Nguyen). The registry is an important tool for the management of GD in France.

Giving patients the opportunity to contribute to the registry is a component of the National Plan for Rare Diseases, and helps to improve knowledge of GD. The inclusion of data in the registry requires the informed consent of the patient [114].

Data from the RFMG have been used in numerous collaborative studies, which have been published in biomedical journals [4, 25, 79, 115–117]. The registry has also made it possible to study the epidemiology of GD in France.

## Appendix 3: The national collection of GD biological samples (ELODIE-MG)

Actions to promote the development of research on rare diseases have been supported since the introduction of the second PNMR (PNMR 2011–2016). Establishing a registry to collect clinical/biological data and creating a collection of biological samples for longitudinal follow-up are essential for research purposes. In this context, the CETG created a national collection of biological samples for GD to facilitate the ELODIE-MG longitudinal study.

Within the framework of this PNDS, the CETG is making all practitioners aware of the collective importance of giving every patient diagnosed with GD (or their parents or legal guardians) the opportunity to contribute to the national collection of GD biological samples. No additional blood tests are required as any surplus remaining from samples taken for tests performed as part of the routine management of the patient can be retained for research purposes instead of being destroyed. The inclusion of samples in the national collection requires the informed consent of the patient [114].

In 2022, four GD reference biomedical laboratories (Paris, Toulouse, Lyon and Clermont-Ferrand) were participating in the collection of samples. The collection is coordinated by the Center for Biological Resources (CRB) in Auvergne and use of the samples is overseen by the CETG, chaired by Dr F. Camou.

## Appendix 4: Examinations to be carried out over the disease course (diagnosis and monitoring)

See Tables 1, 2 and 3.

Depending on the evolution and the clinical presentation, additional examinations may be indicated (see section Initial assessment/Imaging evaluations).

## Appendix 5. Specific treatments for GD

**Table:**

Molecule	Usual dosage	Marketing authorization			
		GD1 (adult)	GD1 (child)	GD2	GD3
Imiglucerase	60 U/kg/every 14 days	YES	YES	NI	YES
Velaglucerase alpha	60 U/kg/every 14 days	YES	YES	NI	NI
Eliglustat^a^ 1 capsule = 84 mg)	SM: 1 capsule × 1/day IM: 1 capsule × 2/day RM: 1 capsule × 2/day URM or undetermined: NI	YES	NI	NI	NI
Miglustat 1 capsule = 100 mg	1 capsule × 3/day (gradual dose increases after initiation are advised)	YES	NI	NI	NI

^a^ The dosage of eliglustat varies according to the CYP2D6 metabolism profile of the patient: SM: slow metabolizer; IM: intermediate metabolizer; RM: rapid metabolizer, URM: ultra-rapid metabolizer. NI: not indicated. For pediatric patients, Cerdelga is indicated for patients with GD1 who are 6 years and older with a minimum body weight of 15 kg, who are stable on enzyme replacement therapy (ERT).

These treatments are prescribed and available exclusively for patients under hospital management (although the treatments can be prescribed for at-home administration).

*Treatment with enzyme replacement Therapy (ERT)*

**Indication, dosage, side effects:** see the Summary of Product characteristics (SmPC) documents for imiglucerase [53] and velaglucerase alpha [54].

**Notes**

- According to the literature, the efficacy and safety of imiglucerase and velaglucerase alpha in GD1 are similar, both in adults and in children [118, 119].
- Only imiglucerase has marketing authorization for GD3.
- An implantable catheter may be required in case of difficulty obtaining peripheral venous access.
- Home administration, supervised by a health care professional, can be considered for patients who have received at least three infusions in hospital with no adverse effects. It is imperative to ensure that the cold chain is maintained when storing the vials. For community-based nurses, prior training in preparing and administering ERT is required. After appropriate training, it is possible for patients to carry out self-infusion, or for a family member to administer the treatment.
- It is recommended that injectable solutions be administered via a 0.2-µm filter.
- For adult patients, the dosage may be reduced to 45 U/kg and then to 30 U/kg every 2 weeks when therapeutic goals have been achieved.
- Increasing the dosage above 60 U/kg every 2 weeks has not been shown to be beneficial in case of worsening disease or for GD3 [14, 81].
- The usual interval between infusions is 2 weeks, a period over which detectable intra-cellular activity of the therapeutic enzyme has been shown to be maintained [120]. In certain cases, it is possible to increase the interval between infusions to every 3 weeks [121–124].
- Any change in dosage or the spacing of infusions must be preceded by an assessment of the disease status of the patient, and followed by quarterly monitoring for 1 year. Assessments should include clinical examinations and biological marker analyses, and imaging evaluations, if necessary, at 6 months and/or 1 year. Any other changes must be discussed during a multidisciplinary consultation meeting.
- Anti-imiglucerase and anti-velaglucerase alpha antibodies may appear (5–15% of cases): they are exceptionally neutralizing [125–127]. Routine testing for these antibodies is not recommended. However, analyses should be considered in the event of allergic reactions or when treatment is ineffective.

*Substrate reduction treatment (SRT)*

**Eliglustat:**

***Indication, dosage, ***side*** effects*****:** see the eliglustat SmPC [56]

***Notes***

Consumption of grapefruit (in any form) carries a risk of overdose through inhibition of CYP3A4 activity and should be avoided. St. John's wort (CYP3A4 inducer) is associated with decreased eliglustat activity. Many drug interactions can be expected with drugs that are metabolized by the same metabolic pathways as eliglustat, and with drugs that modify the activity of enzymes in these pathways. It is useful to get advice from a pharmacologist or a pharmacist if in doubt about any potential drug interactions.

**Miglustat**

***Indication, dosage, side effects:*** see the miglustat SmPC [55]

## Appendix 6: Reference and competence center contact details

### Reference and competence centers for lysosomal diseases (2023 certification)

- *Coordinating Center*

Dr Bénédicte HERON

Department of Neuropediatrics

Trousseau Hospital, 75571 Paris Cedex 12

- *Constituent Centers*

Dr Yann Nguyen

Department of Internal Medicine

Beaujon Hospital, 92110 Clichy

Dr Anaïs BRASSIER

Department of Hereditary metabolic diseases

Necker Hospital, 75015 Paris

Dr Yann NADJAR

Department of Neurology and Neuro-Metabolism

Hospital Pitié Salpêtrière, 75013 Paris

Pr Dominique GERMAIN

Department of Medical Genetics

Raymond Poincaré Hospital, 92380 Garches

Dr Olivier LIDOVE

Department of Internal Medicine

Hôpital de la Croix Saint Simon,75020 Paris

Dr Stéphanie TORRE

Neonatal Pediatrics and Resuscitation

Charles Nicolle University Hospital, 76000 Rouen

- *Competence *Centerslis

Prof. Marc BERGER

Department of Biological Hematology

Estaing University Hospital, 63003 Clermont-Ferrand Cedex

Dr Bérengère CADOR

Department of Internal Medicine

Pontchaillou University Hospital 35033 Rennes Cedex 9

Dr Francis GACHES

Department of Internal Medicine

Clinique Monié , 31290 Villefranche-de-Lauragais

- Secretariat of the CRML and the CETG

**Ms Samira ZEBICHE**

Tel: +33 (0)1 40 87 52 86 or +33 (0)6 77 71 41 59

Fax: +33 (0)1 40 87 44 34

E-mail: samira.zebiche@aphp.fr

Website: www.cetl.net

## Appendix 7: Rare clinical symptoms of GD

- Gallbladder manifestations: a high incidence of biliary lithiasis has been reported in GD [128].
- Cardiac manifestations: reports of heart murmur related to heart valve disease and, much more rarely, chest pain associated with pericarditis (described as hemorrhagic or constrictive in some cases) [129].
- Skin manifestations: yellow–brown pigmentation, predominantly on the face and shins [130].
- Dental symptoms: related to mandibular infiltration and including pseudocysts, and periodontal disease [93].
- Gastrointestinal symptoms (very rare): diarrhea and abdominal pain have been reported, leading to investigations for suspected protein-losing enteropathy, mesenteric adenopathy, ileal lymphoid hyperplasia, digestive hemorrhage, or colon infiltration [131–134].
- Hepatic manifestations: hepatic cytolysis, steatosis, and cirrhosis [135].
- Metabolic manifestations: development of insulin resistance [136].
- Ophthalmological manifestations: opaque corneas and thinning of the retina [137].
- Pulmonary manifestations: cough and dyspnea related to interstitial lung disease, restrictive syndrome secondary to spinal deformities, and PAH, especially in patients who have undergone splenectomy [35].
- Renal manifestations: kidney failure or nephrotic syndrome have been reported in exceptional cases, although it remains uncertain whether these findings were directly associated with GD [138].
- Salivary anomalies (very rare): reduction in saliva secretion [139].

## Appendix 8: Clinical contexts in which a diagnosis of GD should be suspected

Delayed diagnosis of GD is common and can lead to complications [140–143]. In the absence of evidence of any other etiology after carrying out a complete clinical examination, a diagnosis of GD must be systematically considered, and glucocerebrosidase activity measured, in the following situations:

- Before performing a splenectomy (or splenic biopsy).
- Unexplained splenomegaly. The combination of splenomegaly and thrombocytopenia is a primary but non-specific symptom of GD [144].
- Unexplained anemia and thrombocytopenia, especially during pregnancy.
- Immune thrombocytopenia that is resistant to treatment or associated with splenomegaly.
- Hyperferritinemia associated with unexplained thrombocytopenia and/or splenomegaly.
- Hypergammaglobulinemia associated with thrombocytopenia.
- A monoclonal spike associated with unexplained splenomegaly and/or thrombocytopenia.
- Avascular osteonecrosis, bone infarcts, or unexplained bone pain.
- Supranuclear oculomotor palsy (GD3).
- PD (or a parkinsonian syndrome) associated with thrombocytopenia and/or splenomegaly, especially in a young person.

## Appendix 10: Education about treatment, patient rights, and patient associations

It is essential to involve the patient in their care. This approach involves providing information to patients and their carers about:

- The disease, its clinical presentation, and warning signs that should lead to a consultation.
- The way in which the disease is transmitted (genetic counseling).
- Ways of delivering and administering treatments.
- Recognizing adverse drug reactions.
- The importance of compliance.
- Everyday life (planning parenthood, updating vaccinations in case of splenectomy, organizing infusions at home: storing material and enzyme therapy, waste management). Extended travel (holidays, business trips abroad, etc.) must be planned several weeks in advance in order to organize the administration of ERT during the patient’s absence (making contact with the local hospital pharmacy, care provider, etc.).
- Their rights (designation of GD as a chronic health condition, home delivery of enzyme therapy and infusion equipment, disability, sick leave, and access to psychological support).
- Organizing and anticipating administrative procedures (applications to regional departments for persons with a disability: Maison départementale des personnes handicapées, guardianship etc.).
- Preparing the transition of care from pediatric care to adult medicine.

Health care professionals and patients should be informed of the existence of **reference/competence centers** for metabolic diseases, **institutional websites** (HAS [145], Committee for the Evaluation and Treatment of Lysosomal Diseases [146], etc.) and the Orphanet platform [147], where they can find useful information to help them better understand the disease and its management.

The patient association **Vaincre les Maladies Lysosomales** [148], 2 ter avenue de France, 91,300 MASSY (Tel: 0033 1 69 75 40 30), contributes to improving overall provision of disease care by facilitating cooperation between patients, patient associations, and care providers.

There are **therapeutic patient education** programs in which patients with GD can participate (contact: samira.zebiche@aphp.fr).

## Appendix 11: Emergency cards and emergency information fact sheets

Emergency cards are distributed to patients with a rare disease living in France to improve the coordination of their care in emergency situations. These personalized cards are the property of the patients. They were set up as part of the third PNMR (PNMR 2018–2022) by the Direction Générale de l'Offre de Soins (General Directorate for Health care Provision). The cards are confidential. They should be filled in with the collaboration of the physician in charge of the patient’s follow-up.

They include the following information: symptoms to be considered when assessing the patient in an emergency, procedures to be avoided or used in preference in these situations, contact details of the physician and medical center in charge of the patient’s care, and contact numbers. Links to useful information should also be included.

**The emergency cards** were developed and validated through a collaborative process involving the French Ministry of Health, expert networks, and patient associations. The structure of the card is similar for all rare diseases. The "credit card" format allows it to be stored in the patient's wallet or card holder, so that it can be easily consulted in an emergency.

In 2022, emergency cards with relevance for patients with GD had been developed for:

- **Lysosomal Diseases**
- **Inherited Metabolic Disorders**

The emergency cards are available to health care professionals and patients on the G2M website [149].

In addition, a series of emergency information sheets for physicians and emergency service personnel have been developed by ORPHANET for GD1 and GD3, in collaboration with the CETG, VML and the French Society of Emergency Medicine (Société Française de Médecine d'Urgence). These documents provide information about the disease and the main complications of GD that may require urgent medical attention. These emergency information sheets are available on the ORPHANET [147] and G2M websites [149].

## Abbreviations

CCL18: C–C Motif Chemokine Ligand 18
CETG: *Comité d'Evaluation du Traitement de la maladie de Gaucher* (Committee for the Evaluation and Treatment of Gaucher Disease)
*CHIT1*: Chitotriosidase Gene
CRML: *Centre de Référence des Maladies Lysosomales* (Reference Center for Lysosomal Diseases)
CYP2D6: Cytochrome P450, Family 2, Subfamily D, Member 6
ELODIE-MG: *Etude LOngitudinale des Déterminants Intrinsèques et Extrinsèques de la Maladie de Gaucher* (Longitudinal Study of Intrinsic and Extrinsic Determinants of Gaucher Disease)
ERT: Enzyme Replacement Therapy
*GBA1*: Glucocerebrosidase Gene
GD: Gaucher Disease
GD1: Gaucher Disease Type 1
GD2: Gaucher Disease Type 2
GD3: Gaucher Disease Type 3
G2M: Rare Inherited Metabolic Disorders Network
HAS: *Haute Autorité de Santé* (French National Health Authority)
LysoGL1: Glucosylsphingosine
MRI: Magnetic Resonance Imaging
PAH: Pulmonary Arterial Hypertension
PD: Parkinson’s Disease
PNDS: *Protocole National De Diagnostic et de Soins* (French National Protocol for Diagnosis and Care)
PNMR: *Plan National Maladies Rares* (French National Rare Disease Plan)
PSAP: Prosaposin
PT: Prothrombin Time
RFMG: *Registre Français de la Maladie de Gaucher* (French Gaucher Disease Registry)
RFT: Respiratory function test
SmPC: Summary of Product Characteristics
SRT: Substrate Reduction Therapy
VML: *Vaincre les Maladies Lysosomales* (French Patient Association for Lysosomal Diseases)
